# Detecting Prediabetes and Diabetes: Agreement between Fasting Plasma Glucose and Oral Glucose Tolerance Test in Thai Adults

**DOI:** 10.1155/2015/396505

**Published:** 2015-08-06

**Authors:** Wichai Aekplakorn, Valla Tantayotai, Sakawduan Numsangkul, Wilarwan Sripho, Nutchanat Tatsato, Tuanjai Burapasiriwat, Rachada Pipatsart, Premsuree Sansom, Pranee Luckanajantachote, Pongpat Chawarokorn, Anek Thanonghan, Watchira Lakhamkaew, Aungsumalin Mungkung, Rungnapa Boonkean, Chanidsa Chantapoon, Mayuree Kungsri, Kasetsak Luanseng, Kornsinun Chaiyajit

**Affiliations:** ^1^Department of Community Medicine, Faculty of Medicine Ramathibodi Hospital, Mahidol University, Bangkok 10400, Thailand; ^2^School of Nursing, Walailak University, Tha Sala, Nakhon Si Thammarat 80161, Thailand; ^3^Khon Buri Hospital, Khon Buri District, Nakhon Ratchasima 30250, Thailand; ^4^Nakhon Ratchasima City Municipality, Nakhon Ratchasima 30000, Thailand; ^5^Tha Sala Hospital, Tha Sala District, Nakhon Si Thammarat 80160, Thailand; ^6^Buddhachinaraj Hospital, Muang District, Phitsanulok 65000, Thailand; ^7^Samutsakhon Hospital, Muang District, Samutsakhon 74000, Thailand; ^8^Thatphanom Crown Prince Hospital, Thatphanom District, Nakhon Phanom 48110, Thailand; ^9^Pakplee Hospital, Pakplee District, Nakhon Nayok 26130, Thailand; ^10^Nonghualing Health Center, Pakplee District, Nakhon Nayok 26130, Thailand; ^11^Phrae Hospital, Muang District, Phrae 54000, Thailand; ^12^Wang Wiset Hospital, Wang Wiset District, Trang 92220, Thailand

## Abstract

*Aim*. To evaluate an agreement in identifying dysglycemia between fasting plasma glucose (FPG) and the 2 hr postprandial glucose tolerance test (OGTT) in a population with high risk of diabetes. *Methods*. A total of 6,884 individuals aged 35–65 years recruited for a community-based diabetes prevention program were tested for prediabetes including impaired fasting glucose (IFG) or impaired glucose tolerance (IGT), and diabetes. The agreement was assessed by Kappa statistics. Logistic regression was used to examine factors associated with missed prediabetes and diabetes by FPG. *Results*. A total of 2671 (38.8%) individuals with prediabetes were identified. The prevalence of prediabetes identified by FPG and OGTT was 32.2% and 22.3%, respectively. The proportions of diabetes classified by OGTT were two times higher than those identified by FPG (11.0% versus 5.4%, resp.). The Kappa statistics for agreement of both tests was 0.55. Overall, FPG missed 46.3% of all prediabetes and 54.7% of all diabetes cases. Prediabetes was more likely to be missed by FPG among female, people aged <45 yrs, and those without family history of diabetes. *Conclusion*. The detection of prediabetes and diabetes using FPG only may miss half of the cases. Benefit of adding OGTT to FPG in some specific groups should be confirmed.

## 1. Introduction

Identification of individuals with a high risk of developing diabetes and early diagnosis of diabetes are worthwhile for further prevention and early treatment of diabetes, respectively. Effective lifestyle and pharmacologic interventions to delay development of diabetes in people with high risk have been established [1]. Whether screening of diabetes in the general population is cost-effective or improves health outcomes compared with routine clinical diagnosis remains inconclusive. Simmons and colleagues [2] reported findings from ADDITION study which showed no significant difference in all-cause mortality between the screening and control groups. However, screening for prediabetes and diabetes among high risk population with appropriate intervention is more effective than screening for diabetes alone [3].

Thailand has experienced an increasing trend of diabetes over the past two decades and nearly a third of the people with diabetes are undiagnosed [4, 5]. To detect dysglycemia and diabetes, the ADA recommends at least 2 alternatives, namely, fasting plasma glucose (FPG) and 2 hr postprandial oral glucose tolerance test (OGTT) for detection of prediabetes and diabetes [6] with different pros and cons in practice [7]. Normally, in a health care setting, FPG is used to identify diabetes among high risk groups because of the convenience and low cost compared with OGTT. Although OGTT has disadvantages in feasibility for mass screening, it is more sensitive than FPG to identify prediabetes and diabetes. Previous studies reported a high degree of discrepancy in the classification of the two criteria and using FPG missed a lot of cases with diabetes [8]. The extent of OGTT over FPG had not been evaluated in the Thai population. It is interesting and useful to explore which population was missed most by FPG. The objective of this study was to evaluate and document the degree of agreement between FPG and OGTT in the population with a high risk of diabetes. We also examined factors, such as age, sex, or being hypertensive or obese, and explored whether they are associated with undetected prediabetes and diabetes by FPG.

## 2. Methods

In a campaign to enroll candidates with a high risk of diabetes in a community-based experimental study for diabetes prevention program (DPP), 68 primary medical care centers (PC) in 8 study provinces participated in the study during January 2013–August 2013. A community-based screening program was conducted in the community at each PC center. A total of 11,449 participants aged 35 to 65 years who had no previous diagnosis of diabetes but having one or more CVD risk factors joined a screening program for recruitment of impaired glucose tolerance test (IGT) subjects into the community-based DPP study. Pregnant women and patients with known diabetes or cardiovascular diseases were not included in the study. All the participants agreed and signed informed consent to participate in the study. The study was approved by the Ethical Review Board of the Ministry of Public Health.

Criteria used in the recruitment process were as follows: individuals with one of the following risk factors were invited to participate: aged 35 to 65 years, BMI ≥25 kg/m^2^ or abdominal obesity, having history of hypertension, or having a history of diabetes in their siblings or parents. A population-based field screening program was conducted. Individuals who met the criteria were provided with a written instruction to fast overnight. On the test date, the participants were asked about the time they started to fast; if they had not fasted for at least 8 hrs, no blood samples were obtained and the participants were asked to revisit the next day. Two blood samples were obtained from each participant who fasted for at least 8 hours before the scheduled test. The first sample was obtained in the morning at the PC center. After the first sample collection, each participant ingested 75 gm of glucose solution under direct observation by research nurses to ensure compliance of ingestion or any side effects that occurred. Subsequently, the participant laid down in the waiting area and a second blood sample was collected at 120 minutes after the ingestion. The samples were transferred to the community hospital laboratory for analysis of plasma glucose using the hexokinase method. To ensure the comparability, all the laboratories were audited and calibrated with standard samples from the central lab in the Department of Medical service, Ministry of Public Health.

Information on age, sex, previous history of diagnosis of hypertension, and family history of diabetes in siblings or parents was collected using questionnaire interview. Height and weight were measured using a standard technique. BMI was calculated as weight in kilograms divided by the square of height in meters.

### 2.1. Definition of Prediabetes and Diabetes

An individual was classified according to FPG and OGTT as follows: prediabetes was defined as FPG between 100 and <126 mg/dL (impaired fasting plasma glucose, IFG) or impaired glucose tolerance test (IGT) as 2 hr postload glucose (2 hr PG) between 140 and <200 mg/dL. Diabetes was defined as FPG ≥126 mg/dL or 2 hr PG ≥200 mg/dL [6]. Individuals with a previous diagnosis of diabetes were not included in the screening program. For a person who fell into two categories, the higher degree category was applied.

### 2.2. Statistical Analysis

Continuous data are presented as mean and standard deviation (SD). Comparison between means was calculated using Student's *t*-test. The percentages of people with IFG, IGT, FPG ≥126 mg/dL, and 2 hr PG ≥200 mg/dL were calculated. Participants were classified into normoglycemia, isolated IFG, isolated IGT, both IFG and IGT, FPG ≥126 mg/dL alone, 2 hr PG ≥200 mg/dL, and both FPG ≥126 and 2 hr PG ≥200 mg/dL combined. Agreement of participants classified by two criteria was evaluated by Kappa statistics.

The Stata program (Stata version 11.0, Texas, USA) was used for data analysis. The proportion of missed prediabetes by FPG was calculated as number of cases with 2 hr PG between ≥140 and <200 mg/dL but FPG <100 mg/dL divided by total cases of prediabetes and vice versa for the proportion of cases missed by OGTT. The proportion of missed diabetes by FPG was calculated as number of diabetes detected by OGTT (2 hr PG ≥200 mg/dL) but FPG <126 mg/dL divided by the total cases of diabetes. Sensitivity and specificity for detection of prediabetes and diabetes by FPG and OGTT were calculated for all subjects and stratified by subgroups. Logistic regression was used to examine factors associated with missed prediabetes and diabetes by FPG and by OGTT in a separate model. Independent variables included age group (<40, 40–59, and ≥60 yrs), sex, BMI category (<25, 25-<30, and ≥30 kg/m^2^), history of hypertension (yes/no), and family history of diabetes (yes/no). For the model to assess factors related to the missed prediabetes by FPG or OGTT, those participants with diabetes (by FPG or OGTT) were excluded from the model. The odds ratio and 95% confidence interval (95% CI) are reported. Statistical significant level was set at *P* < 0.05. A total of 11,449 individuals participated in the tests and 6,884 (60.1%) were available with complete data. The demographic characteristics including age and sex of those participated were similar to those with complete data in the present study.

## 3. Results

Table 1 shows the characteristics of the participants who attended the screening tests. The mean age (SD) was 50.5 (6.9) years with 69.9% of the subjects aged 45–59 years. 23.6% of the subjects were men. Mean BMI (SD) was 26.2 (4.5) kg/m^2^ with a larger BMI in women than in men (*P* < 0.001). FPG was significantly higher in men than in women (<0.001). 22.3% of the subjects had hypertension and 41.8% had a family history of diabetes in their first degree relatives.

Table 2 shows agreement of classification between FPG and OGTT. A total of 3,487 (50.6%) were positive for dysglycemia defined by either FPG or OGTT. There was poor agreement between the classification of prediabetes and diabetes defined by FPG and OGTT (Kappa value, 0.55). There were 2671 (38.8%) individuals classified as prediabetes. The prevalence of IGT was higher than that of IFG (32.2% versus 25.3%, resp., *P* < 0.05). Isolated IGT was 2.4 times higher than isolated IFG (18.0% versus 7.3%, resp., *P* < 0.05). In addition, diabetes prevalence classified by OGTT was two times higher than those defined by FPG (11.0% versus 5.4%, resp., *P* < 0.001).

Table 3 shows the percentages of prediabetes and diabetes missed by either FPG or OGTT alone. Overall, FPG would miss 46.3% of all prediabetes and 54.7% of all diabetes cases, whereas the corresponding percentages of missed diagnosed by OGTT were 18.9% and 7.0%, respectively. The percentage of missed prediabetes by FPG was higher in women than in men, but the opposite was found for OGTT. Younger individuals (<45 yrs) had higher percentages of missed prediabetes by FPG than in those in the older age group. There was no significant difference in the percentages of missed diagnosis of prediabetes and diabetes by FPG or by OGTT across BMI categories. For detection of prediabetes, 52.3% of those without family history of diabetes were missed by FPG which was higher than their counterpart, but the opposite direction was found for OGTT with a smaller percentage of missed detection. For detection of diabetes, 63.3% of those with hypertension were missed by FPG which was higher than for those without hypertension.  Table 4 shows values of sensitivity and specificity for detection of prediabetes and diabetes by FPG and OGTT. All the specificity values were 100%. The overall sensitivity for detection of prediabetes and diabetes of FPG was 53.7% and 45.4%, respectively, and the corresponding sensitivity of OGTT was 81.1% and 93.0%, respectively. For FPG, the sensitivity for detecting prediabetes was slightly lower among women, younger age group, and those without family history of diabetes, and for diabetes the sensitivity was below 40% among those with hypertension and those with family history of diabetes. For OGTT, all the sensitivity values for prediabetes and diabetes by subgroups were above 75% and 90%, respectively.

Table 5 shows odds ratios for the factors associated with the missed diagnosis of prediabetes and diabetes by FPG or OGTT according to sex, age group, body mass index (BMI), history of hypertension, and family history of diabetes. After controlling for other independent variables, for prediabetes, women were more likely to be missed by FPG but less likely to be missed by OGTT as compared with men. Younger individuals with prediabetes were more likely to be missed by FPG. Compared with individuals with normotension, those with hypertension were less likely to be missed for prediabetes by OGTT. Individuals with BMI <25 were more likely to be independently associated with missed detection of prediabetes by FPG. Those with a family history of diabetes were less likely to be missed for prediabetes but more likely to be missed for diabetes by FPG.

## 4. Discussion

Among people with a high risk for developing diabetes, using OGTT could identify twice as many subjects for both prediabetes and diabetes in addition to the yields from the FPG test. The findings indicate that by using the FPG test alone a substantial number of prediabetes and diabetes cases would be missed. Although FPG is more practical and less expensive compared with OGTT, the latter might be of greater utility in the detection of prediabetes and diabetes. The percentages of missed diagnosis of diabetes by FPG were relatively uniform in some subgroups according to sex, age groups, and BMI categories but more likely to be missed for those with family history of diabetes. Prediabetes might be more likely to be misdiagnosed by FPG among women, those aged <45 yrs, and those without family history of diabetes, indicating the performances of FPG in detecting prediabetes and diabetes were even lower among these subgroups.

The findings of this study are in line with other studies of impaired glucose tolerance and impaired fasting glycemia [9]. The prevalence of IFG was higher in men than in women, but IGT prevalence was higher in women than in men. For those with prediabetes, women and the younger age group were more likely to be missed by the FPG test. For diabetes, those with hypertension were more likely to be missed than those without hypertension. Studies of the performance of FPG in detection of IFG and diabetes compared to the OGTT have been reported in several populations with the findings of higher sensitivity of OGTT compared with FPG. In Europe, Lindahl et al. reported that 31% of patients with diabetes had a normal FPG but an abnormal 2 hr glucose level and only 19% of women and 13% of men having IFG were also classified as IGT [10]. A study of 1554 elderly subjects at high risk of diabetes in Europe found that 16.6% of prediabetes or diabetes was detected by FPG compared to 41.3% detected by OGTT [11]. A recent study in Irish adults also reported that using FPG as initial screening test underestimates the prevalence of prediabetes and diabetes [12].

Both IFG and IGT are intermediate steps preceding diabetes. The conversion of IFG and IGT to diabetes was comparable [13]. Gerstein et al. reported that the estimated annual risk of progression from IGT and/or IFG to diabetes is 5% to 10% [13]. The relative risk (RR) for developing diabetes associated with IGT alone, IFG alone, and both IFG and IGT combined was 5.5, 7.5, and 12.1, respectively. Either IFG or IGT is associated with a 20% increase in risk of CVDs compared with normoglycemia (RR = 1.2) [14]. Microvascular complication was also associated with both IFG and IGT. Compared with individuals with normoglycemia, those with IFG had a higher prevalence of neuropathy [7]. With respect to early detection, the identification of the conditions would provide an opportunity for intervention to prevent organ damage.

FPG and OGTT glucose represent different entities in impaired glucose regulation. IGT is substantially associated with insulin resistance, whereas impaired fasting glucose (IFG) is related to impaired insulin secretion [9]. Although OGTT is more sensitive for detection of dysglycemia, it is less practical and more expensive for use in the population and clinical screening. OGTT is also known for its low reproducibility compared with FPG. The intraindividual coefficients of variation for FPG were 6.4% to 11.4%, whereas for OGTT they were 14.3% to 16.7% [15, 16]. However, in this aspect, a study also found a poor correlation between two serial FPG tests [17]. In a general hospital of the present study province, OGTT was generally applied to those with high risk suggesting that the OGTT is feasible in hospital setting where the physician in charge is aware of the higher sensitivity of OGTT and manages to have the test done. Further studies might be needed to look into the feasibility of other facilities and factors to enable the practice.

The combination of screening for dysglycemia and type 2 diabetes is likely to be more cost-effective than screening for diabetes alone [3]. As women were likely to be missed, OGTT might increase the sensitivity of the detection of prediabetes cases. For those with hypertension, a test of both FPG and OGTT might identify more cases of diabetes. The prediabetes condition leads to a high risk of developing diabetes and confers a high risk of CVD. Identification of prediabetes provides an opportunity of both lifestyle and pharmacological therapy which are effective strategies to avert the progression of diabetes development. Evidence-based lifestyle intervention for impaired glucose regulation is cost-effective [18] and screening for the IFG/IGT conditions and diabetes and intervening with lifestyle interventions are much more cost-effective than screening for diabetes alone and/or without intervention [3]. In countries with affordable resources, utilization of FPG and OGTT for screening of IGT and diabetes among high risk groups may be practical and useful. A further cost-effectiveness study to confirm this might be warranted. Some limitations in the present study included generalization. The data were from people with a high risk; thus it does not reflect the prevalence of IGT and IFG in the general population and might have limited applicability to other populations. However, as the Thai population is at high risk of prediabetes and diabetes, the early detection of people with high risk would be helpful to prevent them from developing diabetes in the near future.

In conclusion, among individuals with a high risk of diabetes, OGTT, in addition to FPG, could identify twice as many individuals with prediabetes and diabetes. The OGTT might be considered to be used in a setting where performing the test is feasible. A combination of both FPG and OGTT might be appropriately tested in some specific groups such as women, age <45 yrs, and those with high blood pressure or family history of diabetes. Application of OGTT in these specific groups should be confirmed, especially in the primary care setting.

## Figures and Tables

**Table 1:** Basic characteristics of participants of the screening program for fasting plasma glucose (FPG) and oral glucose tolerance test (OGTT).

	Men (*n* = 1624)	Women (*n* = 5260)	*P* value^*∗*^
Number			
Age mean (SD) yr	51.5 (6.9)	50.2 (6.8)	<0.001
Age			
<45	20.7	23.4	<0.001
45 to <60	69.8	69.9	
≥60	9.5	6.6	
BMI mean (SD) kg/m^2^	25.3 (4.4)	26.5 (4.5)	<0.001
BMI category kg/m^2^			
<25	50.9	40.5	<0.001
25–29.9	35.2	39.5	
≥30	13.8	20.0	
Hypertension (%)	23.5	22.0	0.21
Family history of diabetes (%)	43.0	41.4	0.33
FPG (mg/dL)	98.4 (27.0)	95.7(23.8)	<0.001
2 hr PG (mg/dL)	146.4 (60.6)	143.3 (56.2)	0.06

^*∗*^ *P* value for comparison between men and women.

**Table 2:** Number (percentage) of participants according to glycemic status classified by FPG and OGTT.

	2 hr PG < 140 mg/dL	2 hr PG 140–<200 mg/dL	2 hr PG ≥ 200 mg/dL	*P* value^*∗*^
FPG < 100 mg/dL	3397 (49.3)	1236 (18.0)	140 (2.0)	<0.001
FPG 100–<126 mg/dL	505 (7.3)	930 (13.5)	306 (4.4)	
FPG ≥ 126 mg/dL	3 (0.04)	54 (0.8)	313 (4.5)	
Total	3905 (56.7)	2220 (32.2)	759 (11.0)	

^*∗*^ *P* value from Chi-square test for all categories.

**Table 3:** Percentages of prediabetes and diabetes missed by FPG and OGTT.

	All prediabetes	% prediabetes, missed by FPG	% prediabetes, missed by OGTT	All diabetes	% diabetes missed by FPG	% diabetes missed by OGTT
All (*n* = 6884)	2671	1236 (46.3)	505 (18.9)	816	446 (54.7)	57 (7.0)
Sex						
Men	618	235 (38.0)^*∗∗*^	153 (24.8)^*∗∗*^	232	127 (54.7)	24 (10.3)^*∗*^
Women	2053	1001 (48.8)	352 (17.1)	584	319 (54.6)	33 (5.6)
Age group (yr)						
20–<45	481	248 (51.6)^*∗∗*^	85 (17.7)	170	91 (53.5)	11 (6.5)
45–<60	1848	842 (45.6)	373 (20.2)	544	304 (55.9)	39 (7.2)
≥60	220	86 (39.1)	36 (16.4)	97	48 (49.5)	6 (6.2)
Hypertension						
Yes	661	301 (45.5)	120 (18.1)	237	150 (63.3)^*∗∗*^	9 (3.8)^*∗*^
No	2010	935 (46.5)	385 (19.1)	579	296 (51.1)	48 (8.3)
Family history of diabetes						
Yes	821	327 (39.8)	199 (24.2)^*∗*^	311	200 (64.3)^*∗*^	18 (5.8)
No	952	498 (52.3) ^*∗∗*^	186 (19.5)	308	173 (56.2)	18 (5.8)
BMI (kg/m^2^)						
<25	919	441 (48.0)	195 (21.2)	242	137 (56.6)	18 (7.4)
25–<30	963	451 (46.8)	174 (18.1)	302	171 (56.6)	21 (6.9)
≥30	519	225 (43.3)	85 (16.4)	185	100 (54.1)	10 (5.4)

All prediabetes: IFG (FPG, 100–<126 mg/dL) or IGT (2 hr PG: 140–<200 mg/dL) or both; all diabetes: FPG ≥126 mg/dL or 2 hr PG ≥200 mg/dL or both, ^*∗*^ *P* value < 0.05, ^*∗∗*^ *P* value < 0.01.

**Table 4:** Sensitivity and specificity for detecting prediabetes and diabetes by FPG and OGTT stratified by selected factors.

	Prediabetes by FPG		Prediabetes by OGTT		Diabetes by FPG		Diabetes by OGTT	
	(*n* = 6068)		(*n* = 6068)		(*n* = 6884)		(*n* = 6884)	
	Sensitivity (%)	Specificity (%)	Sensitivity (%)	Specificity (%)	Sensitivity (%)	Specificity (%)	Sensitivity (%)	Specificity (%)
All cases	53.7	100	81.1	100	45.4	100	93.0	100
Sex								
Men	62.0	100	75.2	100	45.3	100	89.7	100
Women	51.2	100	82.8	100	45.4	100	94.3	100
Age group (yr)								
20–<45	48.4	100	82.3	100	46.5	100	93.5	100
45–<60	54.4	100	79.8	100	44.1	100	92.8	100
≥60	60.9	100	83.6	100	50.5	100	93.8	100
Hypertension								
Yes	54.5	100	81.8	100	36.7	100	96.2	100
No	53.5	100	80.8	100	48.9	100	91.7	100
Family history of diabetes								
Yes	60.2	100	75.8	100	35.7	100	94.2	100
No	47.7	100	80.5	100	43.8	100	94.2	100
BMI (kg/m^2^)								
<25	52.0	100	78.8	100	43.4	100	92.6	100
25–<30	53.2	100	81.9	100	43.4	100	93.1	100
≥30	56.7	100	83.6	100	45.9	100	94.6	100

Cut-off point for detecting prediabetes by FPG at 100 mg/dL and by OGTT at 140 mg/dL; cut-off point for detecting diabetes by FPG at 126 mg/dL and by OGTT at 200 mg/dL.

**Table 5:** Adjusted odds ratios for factors associated with prediabetes and diabetes missed by FPG and OGTT.

	*n*	Odds ratios (95% CI)			
		Missed prediabetes, by FPG	Missed prediabetes, by OGTT	Missed diabetes by FPG	Missed diabetes by OGTT
All (*n* = 6884)	6884				
Sex					
Men	1624	1	1	1	1
Women	5260	1.9 (1.5, 2.4)	0.6 (0.4, 0.7)	1.00 (0.7, 1.4)	0.6 (0.3, 1.3)
Age group (yr)					
20–<45	1532	1.6 (1.1, 2.4)	1.2 (0.7, 2.0)	1.1 (0.6, 2.3)	0.8 (0.2, 3.5)
45–<60	4703	1.3 (0.9, 1.8)	1.4 (0.9, 2.3)	1.3 (0.7, 2.3)	1.0 (0.3, 3.5)
≥60+	490	1	1	1	1
Hypertension					
Yes	1538	1.1 (0.9, 1.4)	0.6 (0.4, 0.8)	1.3 (0.9, 1.9)	0.6 (0.2, 1.3)
No	5346	1	1	1	1
Family history of diabetes					
Yes	2220	0.6 (0.5, 0.7)	1.4 (1.1, 1.8)	1.5 (1.1, 2.1)	0.9 (0.4, 1.8)
No	3092	1	1	1	1
BMI (kg/m^2^)					
<25	2649	1.4 (1.1, 1.9)	1.4 (1.0, 1.9)	1.3 (0.8, 2.1)	1.2 (0.4, 3.2)
25–<30	2379	1.3 (1.0, 1.6)	1.1 (0.8, 1.6)	1.2 (0.8, 1.8)	1.3 (0.5, 3.2)
≥30	1149	1	1	1	1

All prediabetes: IFG (FPG, 100–<126 mg/dL) or IGT (2 hr PG: 140–<200 mg/dL) or both; all diabetes: FPG ≥126 mg/dL or 2 hr PG ≥200 mg/dL or both.
